# Evaluation of Urinary Big Endothelin-1 in Feline Spontaneous CKD

**DOI:** 10.3390/ani10112144

**Published:** 2020-11-18

**Authors:** Marco Giraldi, Saverio Paltrinieri, Camilla Piazza, Paola Scarpa

**Affiliations:** 1 Department of Veterinary Medicine, University of Milan, Via dell’Università, 6, 26900 Lodi (LO), Italy; m.giraldi.vet@gmail.com (M.G.); camilla.piazza92@gmail.com (C.P.); paola.scarpa@unimi.it (P.S.); 2 Veterinary Teaching Hospital, University of Milan, Via dell’Università, 6, 26900 Lodi (LO), Italy

**Keywords:** chronic renal failure, hypertension, proteinuria, urine

## Abstract

**Simple Summary:**

Chronic kidney disease is a common and progressive disease of elderly cats. It is a cause of pet suffering and owner expense. Biologic biomarkers for early diagnosis and for noninvasive evaluation of kidney damage are certainly useful in both research and clinical practice. In this study, we evaluated the biomarker big endotelin-1 in the urine of cats affected with chronic kidney disease. Big endothelin-1 is molecule linked to inflammation and pressure regulation, and it was not previously evaluated in nephropathic cats. We found that urinary big endothelin was increased in patients at late stage of the disease and in patients with proteinuria (a marker of kidney damage). Despite that, big endothelin 1 seemed not to be a useful biomarker for disease progression. According to results of this preliminary study, we suggest this biomarker for future research on feline kidney disease.

**Abstract:**

The endothelin-1 (ET-1) system has been implicated in the development and progression of chronic kidney disease (CKD). No information on big ET-1 in feline urine is available. The purpose of this study was to evaluate if urinary big endothelin-1 (bigET-1) is associated with feline CKD. Sixty urine samples were prospectively collected from 13 healthy cats at risk of developing CKD and 22 cats with CKD of different International Renal Interest Society (IRIS) stages (1–4). Urinary bigET-1 was measured using a commercially available ELISA. BigET-1 normalized to urine creatinine (bigET-1:UC) was compared amongst stages and substages, as proposed by IRIS, and correlated with serum creatinine concentration, proteinuria and blood pressure. BigET-1:UC at the time of inclusion was compared between cats that remained stable and cats that progressed after 12 months. BigET-1:UC was significantly higher (*p* = 0.002) in cats at IRIS stages 3–4 (median: 21.9; range: 1.88–55.6), compared to all other stages, and in proteinuric (*n* = 8, median: 11.0; range: 0.00–46.4) compared with nonproteinuric cats (*n* = 38 median: 0.33; range: 0.00–55.6) (*p* = 0.029). BigET-1:UC was not associated with CKD progression. Urinary bigET-1 increased in advanced stages of CKD and in proteinuric patients, suggesting that ET-1 may be indicative of the severity of feline CKD.

## 1. Introduction

Chronic kidney disease (CKD) is the most common metabolic disease in cats, with a prevalence of up to 50% in cats older than 10 years [1]. The underlying aetiology of CKD often remains obscure. The typical histological features of the idiopathic form of feline CKD include interstitial inflammation, fibrosis and renal tubular damage [2]. Other specific causes of CKD include amyloidosis, polycystic kidney disease and congenital disorders [3]. Feline CKD is variably progressive and is associated with proteinuria, hyperphosphatemia, anemia and systemic arterial hypertension [2,4].

Endothelin-1 (ET-1) is a biological active peptide that is synthesized by almost every cell type. It is produced in the extracellular environment by enzymatic degradation of big endothelin-1 (bigET-1), that is in turn derived from preproendothelin-1. Studies in people have demonstrated that ET-1 is related to many pathological mechanisms and diseases; namely systemic inflammation and cardiovascular, pulmonary and renal diseases [5,6]. In people affected with CKD, ET-1 is associated with inflammation, fibrosis, proteinuria and hypertension [7]. ET-1 contributes to the pathological mechanisms underlying CKD [8,9,10] and expression of ET-1 was found to increase in both glomerular and renal tubular cells secondary to ischemic injuries and inflammation [11,12]. Uremic toxins were also shown to increase renal tubular expression of bigET-1 and ET-1 [13].

In humans, plasma and urinary ET-1 are increased in patients with CKD [14]. Additionally, urinary ET-1 correlates with the severity of renal injury in lupus nephritis [15], with the presence of focal segmental glomerulosclerosis [14] and with the magnitude of proteinuria in different renal diseases [16,17]. ET-1 has also been found to be higher in the plasma and urine of people affected with systemic hypertension associated with CKD [18,19]. Similar results were described in dogs, in which the increased blood concentration of ET-1, evaluated indirectly by measuring its precursor bigET-1, was associated with the severity of CKD and with hypertension associated with CKD [20].

ET-1 was evaluated in cats affected by cardiomyopathies, showing that plasma ET-1 was significantly higher in sick patients when compared to healthy patients [21]. Moreover ET-1 was found to be higher in bronchoalveolar lavage fluid of asthmatic cats when compared to healthy cats [22]. However, no such evaluations are available regarding ET-1 or bigET-1 in cats with CKD.

The aims of this study were: (1) to gain preliminary information about the urinary concentration of bigET-1 in cats with CKD and in cats at risk of developing CKD, with or without proteinuria and hypertension; (2) to evaluate whether urinary bigET-1 may predict progression of CKD in a longitudinal study. We hypothesized that the concentration of urinary bigET-1 would be higher in cats affected with CKD, and particularly in cats with concurrent azotemia, proteinuria and hypertension.

## 2. Materials and Methods

### 2.1. Patients

This prospective study was conducted with client-owned cats presented to the Veterinary Teaching Hospital of the University of Milan from November 2014 to May 2017. Informed consent to collect samples for diagnostic purposes was obtained from all owners. According to the ethical committee statement of the University of Milan number 2/2016, the residual volume of the biological samples collected for diagnostic purposes may also be used for research purposes, after completing the tests required by the submitting Veterinarian.

The population of cats included in the current study was composed of healthy cats at risk of developing CKD and cats already affected with CKD. Cats at risk of developing CKD were: (1) cats older than 10 years [1], or (2) Persian and Maine Coon purebred cats of any age, as these breeds have a known predisposition for polycystic kidney disease [23], and a high prevalence of CKD [24] respectively. Cats from both subgroups presented for annual health checks and had unremarkable physical examination results. Systolic blood pressure (SBP), complete blood count, plasma biochemical assessment (see below) and complete urinalysis were performed for all cats. Patients with nonspecific or potentially nonpathological abnormalities in these tests (such as borderline proteinuria or high SBP) were included in the “at-risk” group if values returned to within normal limits at the following check within 6 months (for example, hypertension at the first visit and normotension at the following check).

Cats in the CKD group were of any age and breed; diagnosis was made on the basis of history, physical evaluation and laboratory results. Azotemic CKD was diagnosed where there was a persistent increased serum creatinine above the internal laboratory reference interval (RI: <140 μmol/dL) and concurrent inadequate urinary concentration ability (persistent urine specific gravity (USG) < 1.035). Nonazotemic CKD was diagnosed where serum creatinine was normal (<140 μmol/L) but one or more of the following abnormalities was present: (1) inadequate urinary concentration ability (persistent USG < 1.035), (2) abnormal renal imaging findings or (3) serially increasing serum creatinine concentration (over a time period of >3 months) [25]. These cats were staged in accordance with the International Renal Interest Society (IRIS) staging guidelines [26]. Whenever clinical examination and laboratory tests were suggestive of the presence of disease other than CKD, further specific diagnostic tests were performed. All the cats were part of a prospective study in which patients were monitored and sampled over a period of 18 months after inclusion. Specifically, cats in the at-risk group and IRIS stage 1 cats were checked every 6 months; IRIS stage 2–4 cats were checked every 3 months. The same tests performed at the time of study enrolment were also carried out at each checkpoint (see below).

Stability or progression of CKD was determined with consideration to a period of 12 months. Cats were defined as “stable” when they maintained the same IRIS stage over the whole 12 months; cats were determined to have progressive CKD where there was progression to a higher IRIS stage (for example, from at risk to IRIS 2 or from IRIS 3 to IRIS 4) within 12 months.

The presence of infectious, endocrine or cardiovascular diseases, malignant tumors, acute kidney injury, urinary tract infections or active sediment were considered as exclusion criteria for cats, both at the time of study enrolment and at any checkpoint. Cats treated with drugs affecting blood pressure, diuretics or anti-inflammatory drugs in the 4 weeks before inclusion were also excluded. These exclusion criteria were also applied at any checkpoint. Therefore, follow-up samples of cats treated with drugs to reduce SBP were not included. All cats affected with CKD (at any IRIS stages) received a commercial dry and/or wet renal diet, and this was not considered an exclusion criterion.

### 2.2. Collection of Data and Samples

A complete physical examination was performed for each cat. Systolic blood pressure (SBP) was measured after a period of hospital acclimatization according to published guidelines using a non-invasive doppler technique (Minidop ES-100VX, Hadeco, Kanagawa, Japan).

Blood (3 mL) was collected from each cat without sedation from the cephalic or jugular vein after a 12 h fasting period. Blood was collected into tubes with ethylenediaminetetraacetic acid to perform routine hematology (Sysmex XT 2000 iV, Sysmex, Kobe, Japan) and in tubes without anticoagulants to obtain serum by centrifugation. A panel of biochemical parameters (urea, creatinine, protein, albumin, glucose, cholesterol, triglycerides, aspartate aminotransferase, alanine aminotransferase, γ-glutamyl transferase, alkaline phosphatase, sodium, potassium, calcium, and phosphorus) was measured using an automated spectrophotometer (Cobas Mira, Roche Diagnostic, Basel, Switzerland).

At least 8 mL of urine was collected from each cat without sedation by ultrasound-guided cystocentesis into a syringe and sent to the internal clinical pathology laboratory. A complete urinalysis, including USG, dipstick analysis and sediment evaluation was performed immediately on each sample as previously reported [27]. Urinary protein (UP) and urinary creatinine (UC) to calculate urinary protein:creatinine ratio (UPC) were measured on urine supernatant from all samples. Specifically, UP was measured with the pyrogallol red molybdate method on undiluted supernatant; UC was measured with the modified Jaffe method diluting supernatants 1:20 with distilled water. Both of these tests were performed using an automated biochemical analyzer (Cobas Mira, Roche Diagnostics). Active sediment was defined as >5 white blood cells (WBC) per high power field (hpf, 400×) and >20 red blood cells (RBC) per hpf. Urinary tract infection (UTI) was diagnosed whenever compatible clinical signs and/or detection of pyuria at the microscopic sediment evaluation (i.e., more than 5 WBC/hpf) plus positive urine culture were detected. Active sediment and UTI were exclusion criteria. To fulfil study requirements, the remaining supernatant obtained from the sediment preparation (5 min at 450× *g*) was aliquoted (400 μL) and stored at −20 °C within 4 h from collection. All these tests were performed at the time of inclusion and at each checkpoint.

### 2.3. Measurement of BigET-1

BigET-1 was measured in urine samples using a solid phase sandwich ELISA developed for human bigET-1 (IBL international GmbH, Hamburg, Germany). BigET-1 is preferred to the biologically active ET-1 since the latter has a shorter half-life and the concentration of the two are considered proportional [12]. According to the kit manufacturer, the primary antibody is specific for the C-terminal 22–38 amino acid sequence of bigET-1 and the secondary detecting antibody is directed against a different antigenic site of the molecule, both antibodies were of rabbit origin. The kit used includes a standard solution containing porcine bigET-1. The reported measurement range is 0.78–100 pg/mL.

Samples were analyzed in batches after a maximum period of 18 months of storage. Aliquots were gently thawed at +4 °C overnight and warmed at room temperature one hour before analysis. A partial validation of the kit was performed following the American Society of Veterinary Clinical Pathology (ASVCP) guidelines [28]. To achieve validation, 12 urine samples of cats at different IRIS stages were randomly selected from the study population and assayed in a first session of work. These samples were used to prepare reference materials. Specifically, a “high pool” and a “low pool” were prepared by mixing the samples (*n* = 6) with the highest and the samples (*n* = 6) with the lowest bigET-1 concentration, respectively. The two pools were used to test intra-assay precision and linearity under dilution (LUD). To test intra-assay precision, the 2 pools were tested 5 times each. The mean value and the standard deviation (SD) were calculated using an Excel spreadsheet and employed to calculate the coefficient of variation (CV = SD/mean × 100). The LUD test was performed by serially diluting the “high pool” by a twofold dilution scheme (i.e., 1:2, 1:4 and 1:8) using distilled water as a diluent. Each dilution was tested in duplicate and the mean value between the two duplicates was considered the actual value (obtained value).

All available urine samples, collected either at the time of inclusion or at the following checkpoints, were selected and used for analysis of bigET-1. These stored samples were assayed in two different sessions of work. The ratio between bigET-1 and urinary creatinine (bigET-1:UC), converting urinary creatinine in mg/mL to match for the ratio, was calculated to normalize the bigET-1 concentration to urine dilution. All the procedures were performed following the instructions of the kit manufacturer.

### 2.4. Statistical Analysis

A commercially available software (GraphPad Prism 5.00; Graph-Pad Software, San Diego, CA, USA) was used to perform statistical analysis. A *p* value < 0.05 was considered statistically significant. Distribution of variables was assessed by the Kolmogorov−Smirnov test and non-parametric statistical tests were applied.

For the LUD test, linear regression between expected and observed values was calculated and the percentage of recovery of the observed values compared with expected values at each dilution was also calculated as follows (1):

recovery = mean observed/expected × 100(1)

Using all available results recorded at the first visit or at the following checkpoints, the Mann−Whitney *U* test was used to compare the urinary bigET-1:UC ratio obtained in cats at risk with cats with CKD, in proteinuric cats (UPC ≥ 0.4) with nonproteinuric or borderline proteinuric cats (UPC < 0.4) and in normotensive or prehypertensive cats (SBP < 160 mmHg) with hypertensive or severely hypertensive cats (SBP ≥ 160 mmHg), including results recorded at the first visit and at the following checkpoints. These latter thresholds for UPC and SBP were used because treatment of proteinuria and hypertension, respectively, is clinically indicated above these values according to current guidelines [29,30]. The Kruskal−Wallis test with post hoc Dunn test was used to compare bigET-1:UC ratio recorded in the different IRIS stages and in the different IRIS substages based on the UPC ratio. Since the total sample number from cats of IRIS stage 3 and 4 was low, samples from these IRIS stages were considered as a single group (labeled “IRIS 3–4”). Correlations between bigET-1:UC and serum creatinine, UPC ratio and SBP were assessed with the Spearman correlation test, including results recorded at the first visit and at the following checkpoints. In these analyses, samples collected at the time of inclusion (T_0_) and at the following available checkpoints were included.

For the longitudinal study, the Mann−Whitney *U* test was used to investigate the difference in bigET-1:UC at T_0_ between stable cats and cats with progressive CKD, including patients with at least 2 available samples collected 12 months apart.

## 3. Results

### 3.1. Preliminary Method Validation

The results of the intra-assay precision are shown in Table 1.

**Figure 1:**
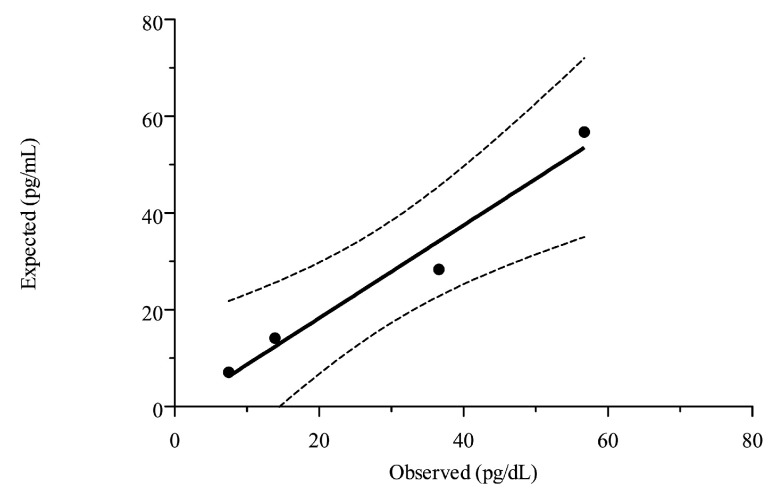
Linearity under dilution of the “high pool” obtained by mixing 6 samples with high big endothelin-1 concentration. The black solid line represents the linear fit between expected and observed data and the black dashed line represents the 95% confidence interval.

The LUD test (Figure 1) demonstrated an excellent degree of linearity (r^2^ = 0.97; *p* = 0.01, slope = 0.96, confidence interval of slope = 0.42–1.04) and the mean ± SD of the recovery rate was 110.7 ± 16.1%.

### 3.2. Samples

Thirty-five cats fulfilled the inclusion criteria and were included in the study (Table 2).

Sixty urine samples collected either at the first visit or during the follow-up checks in the 35 cats were available for analysis. Therefore, more than one sample was collected from some cats. Descriptive statistics of serum creatinine concentration, UPC ratio and SBP, and substages of 60 samples grouped according to the IRIS stages are reported in Table 3.

### 3.3. BigET-1 in the Different Groups and Subgroups

BigET-1:UC was not significantly different between cats in the at-risk group and cats with CKD. However, when samples were grouped according to IRIS stage, bigET-1:UC was significantly higher in IRIS 3-4 group compared to all other stages (Table 4).

A weak but significant correlation was also found between bigET-1:UC and serum creatinine (*p* = 0.007; r = 0.344).

Grouping samples according to proteinuria, bigET-1:UC was significantly higher in proteinuric cats (UPC > 0.4) compared to cats with UPC < 0.4 (*p* = 0.011). Among the 3 substages of proteinuria, bigET-1:UC was significantly higher in proteinuric cats compared with nonproteinuric cats, but not if compared with borderline proteinuric cats (Table 4). No significant correlation (*p* = 0.056) was found between UPC and bigET-1:UC.

No significant difference was found in bigET-1:UC between cats with SBP < 160 mmHg and cats with SBP ≥ 160 mmHg (Table 4). No significant correlation (*p* = 0.793) was found between SBP and bigET-1:UC.

### 3.4. Longitudinal Study

Twenty cats had at least two serum samples collected at the time of inclusion (T_0_) and 12 months later and were included in the longitudinal study. Amongst these cats, the mean ± SD of the follow up was 13.1 ± 3.2 months. Twelve cats were classed as at risk at T_0_, 4 of these cats remained stable and 8 progressed to CKD (4 to IRIS stage 1 and 4 to IRIS stage 2).

Eight cats had a diagnosis of CKD at T_0_, 6 of these cats remained stable (2 at stage 1, 3 at stage 2 and 1 at stage 3) and 2 progressed (1 from stage 2 to stage 3 and 1 from stage 3 to stage 4). Urinary bigET-1:UC did not significantly differ (*p* = 0.909) between cats that remained stable (median, 0.01; range, 0–0.10) and cats that developed CKD (median, 0.12; range, 0–0.09) during the first 12 month after inclusion.

## 4. Discussion

BigET-1 was detected using a commercially available ELISA kit and, to the authors’ knowledge, this is the first study evaluating this peptide in feline urine. Measurement of bigET-1 was preferred to the biologically active metabolite ET-1 since the former has a longer half-life compared to ET-1 and a proportional relationship is assumed between the two peptides [31]. Moreover, the binding between ET-1 and the receptor is quasi-irreversible [12]. The capturing antibody of the ELISA kit used in this study was toward the 22–38 sequence of human bigET-1. According to a previous study, human and feline bigET-1 differ for 2 amino acids in this part of the peptide [32]. Therefore, it is possible that using an ELISA kit specific for feline bigET-1 would enhance the analytical sensitivity. However, the ELISA kit used in this study was able to detect bigET-1 in feline urine.

In our population of cats, urinary bigET-1:UC tended to increase at late stages of CKD, suggesting that this peptide cannot be considered an early biomarker. Late stages of feline CKD are characterized by severe azotemia and often an increasing proteinuria, accompanied by severe interstitial inflammation and fibrosis [2,33]. Interestingly, in humans all these factors have been associated with overexpression of ET-1 [12,13]. Therefore, it is possible that activation of the ET-1 system could also occur in cats affected by CKD, at least in the more severe stages of the disease. However, the actual contribution of this pathway to the pathogenesis of feline CKD, and whether the increase of bigET-1 (and ET-1) is a contributing cause or a consequence of CKD, is still unknown. Despite the increase of bigET-1 at late stages of the disease, no difference was found between the groups of at-risk cats and cats with CKD. The lack of significant difference was probably due to the relatively high number of affected cats with undetectable urinary bigET-1 in the CKD group. Additionally, the correlation between serum creatinine and bigET-1:UC was weak. These results, along with the wide overlap of urinary bigET-1 among the different IRIS stages, limit the value of bigET-1 as diagnostic biomarker for feline CKD in routine clinical practice.

Proteinuric cats demonstrated higher bigET-1:UC compared to nonproteinuric cats. ET-1 synthesis may be part of the inflammatory setting of the tubulo-interstitial damage and/or could be stimulated by protein overload on tubular cells, as demonstrated in vitro [34]. Alternatively, given that proteinuria in cats with CKD is mainly due to tubular damage [35] (at least in the idiopathic CKD) and low molecular proteins and peptides in the ultrafiltrated urine are mainly reabsorbed by tubular cells [36], it is possible to speculate that urinary bigET-1 could increase in proteinuric cats due to reduced resorption by damaged tubular cells. Moreover, at the late stage of CKD ultrafiltrated creatinine is reabsorbed by tubular cells. This phenomenon could affect the ratio between bigET-1 and creatinine and, in turn, could falsely increase bigET-1:UC in severely azotemic patients. The high bigET-1:UC in proteinuric patients could be considered a further confirmation of the involvement of the ET-1 system in feline CKD. However, the wide overlap of bigET-1:UC among the IRIS substages of proteinuria and the lack of significant correlation between bigET-1:UC and UPC limits the ability of this biomarker to identify proteinuric cats.

No association was found between urinary bigET-1 and SBP. Although its pathogenesis is not well clarified [4], systemic hypertension is an important complication associated with CKD. Measurement of SBP and hypertension diagnosis could be complicated by “situational hypertension” in cats; that is, the temporary increase of SBP in a clinical setting, mainly due to stress [37]. Therefore, a biomarker associated with true hypertension in cats could be useful in clinical practice to identify cats requiring further investigation according to the current guidelines for diagnosis and management of systemic hypertension. Similar to other biomarkers previously evaluated [38], our results suggest that urinary bigET-1 cannot be recommended to detect systemic hypertension associated with CKD or to differentiate true hypertension from situational hypertension in cats. Some studies have shown increased urinary concentration of ET-1 in humans affected with systemic hypertension associated with CKD [18,19]. However, urinary ET-1 (and bigET-1) concentration is deemed to depend on the local renal expression instead of systemic expression [39]. Therefore, although the involvement of ET-1 in the pathogenesis of hypertension associated with feline CKD is yet to be demonstrated, the lack of association between urinary bigET-1 and systemic hypertension is not an unexpected result.

Urinary bigET-1 was not predictive of IRIS stage progression after 1 year in the longitudinal study. In humans, ET-1 was associated with disease activity in lupus nephritis and was deemed as a potentially useful biomarker for disease progression [15]. Our results suggest that urinary bigET-1 might be more indicative of the current CKD severity in cats, rather than the future progression of the disease.

This study had several limitations. Firstly, the validation tests were preliminary and it is a limitation of this study. The low intra-assay precision, especially at low concentrations of bigET-1, coupled with the poor recovery rates detected with the LUD test, limit the interpretation of results. BigET-1 concentration in the “low pool” was close to the lower limit of detection and, as a result, imprecision or inaccuracy could be higher at this level. Hence, bigET-1 results should be interpreted with caution at these concentrations, on which, however, the high analytical imprecision has a little consequence on clinical decisions. The poor recovery rates could reflect some matrix effect on the ELISA kit, which in turn could affect the results of the diluted samples. Secondly, measurement of bigET-1 was performed on samples stored at −20 °C, some samples were stored for up to 18 months. Stability of bigET-1 under these storage conditions has not been evaluated and preanalytical reduction of the biomarker is possible. However, the possible reduction over time of the biomarker due to the storage at −20 °C should affect similarly all the study groups and, in turn, should not affect interpretation of the results significantly. It is possible that some cats classified as “at risk” were actually at IRIS stage 1, given the lack of ultrasonography in many cases. Furthermore, the presence of other diseases potentially affecting bigET-1 could not be completely excluded in all the enrolled cats, since laboratory tests aimed to exclude specific diseases were performed only in the presence of compatible clinical signs and/or laboratory results. The presence of borderline proteinuria and hypertension in some cats classified as “at risk” was considered to be secondary to microhematuria and situational hypertension, respectively; however, in these cases it was not possible to definitively exclude the presence of an early stage of CKD or other related diseases. Finally, the low number of enrolled cats and the inclusion of all the samples obtained at any checkpoints in the study groups limits the power of the statistical analysis. However, strict inclusion criteria were applied for this study and this notoriously limits the possibility to recruit high numbers of patients in prospective “in field” studies on owned animals. Therefore, the inclusion of additional cases was deemed not necessary especially due to the preliminary nature of this study, that must be considered as a first investigation on a biomarker previously not evaluated in cats with CKD. Therefore, further studies are warranted to perform a more complete validation of the ELISA kit used in this study, including comparison with a gold standard test, as well as the evaluation of storage stability, and of the biomarker in a wider population of cats.

## 5. Conclusions

In conclusion, although a higher number of cats and samples would be necessary for conclusive evidence, our results suggest that urinary bigET-1 seems indicative of the current CKD severity.

## Figures and Tables

**Table 1:** Results of the five intra-assay repetitions of the “low pool” and the “high pool”, prepared by mixing the 6 samples with the lowest bigET-1 concentration and the 6 samples with the highest bigET-1 concentration.

	1st (pg/mL)	2nd (pg/mL)	3rd (pg/mL)	4th (pg/mL)	5th (pg/mL)	Mean ± SD (pg/mL)	CV (%)
Low pool	6.1	5.3	8.2	2.7	4.9	5.5 ± 1.9	34.5
High pool	51.9	65.7	55.7	57.8	52.3	56.7 ± 5.6	9.9

CV = coefficient of variation; SD = standard deviation.

**Table 2:** International Interest Renal Society (IRIS) stages, gender, breed and age of the 35 cats enrolled.

	At Risk (*n* = 13)	CKD (*n* = 22)
Stage		IRIS 1 = 4 IRIS 2 = 12 IRIS 3 = 4 IRIS 4 = 2
Gender	Male = 7 (1 intact, 6 castrated) Female = 6 (all spayed)	Male = 9 (all castrated) Female = 13 (all spayed)
Breed	DSH = 10	DSH = 15
	Purebred * = 3	Purebred ^†^ = 7
Median age (range)	10.9 (5.5–15.5) years	11.4 (6.0–18.8) years

CKD, chronic kidney disease; DSH = domestic shorthair. * 1 Persian, 1 Exotic and 1 Maine Coon. ^†^ 2 Siberian 1 Norwegian Forest, 1 Persian, 1 Siamese, 1 Chartreux, 1 Exotic.

**Table 3:** Selected clinical and clinicopathologic variables of feline patients grouped according to International Renal Interest Society (IRIS) staging. Samples collected at the time of inclusion and at the different checkpoints were included. Continuous variables were represented as the median (range). Number and percentage (in brackets) of cases included in each IRIS substage are also reported.

Variables	Cats at Risk (*n* = 21)	IRIS 1 (*n* = 9)	IRIS 2 (*n* = 21)	IRIS 3 (*n* = 6)	IRIS 4 (*n* = 3)
Serum creatinine (μmol/L)	120 (68–139)	130 (78–139)	174 (144–391)	312 (281–422)	625 (467–959)
UP (mg/dL)	23.3 (1.0–97.6)	31.8 (5.2–52.9)	18.0 (6.1–105.0)	17.7 (6.6–39.1)	11.0 (4.4–91.3)
UC (mg/dL)	174.2 (50.4–401.4)	196.3 (132.6–510.8)	172.4 (25.9–464.4)	65.7 (27.6–163.2)	32.3 (27.6–45.7)
UPC	0.2 (0.1–0.4)	0.1 (0.1–0.3)	0.1 (0.1–0.8)	0.3 (0.1–0.6)	0.2 (0.2–2.7)
NP	13 (21.7%)	6 (10%)	15 (25%)	3 (5%)	1 (1.7%)
BP	8 (13.3)	3 (5%)	2 (3.3)	−	1 (1.7%)
P	−	−	4 (6.6%)	3 (5%)	1 (1.7%)
SBP * (mmHg)	130 (100–155)	140 (130–200)	145 (110–230)	140 (125–160)	150 (140–160)
NT	14	5	7	3	1
PHT	3	−	2	−	−
HT	2	3	3	2	1
SHT	−	−	1	−	−

BP = borderline proteinuric; CKD = chronic kidney disease; HT = hypertensive; IRIS = International Renal Interest Society; NP = nonproteinuric; NT = normotensive; P = proteinuric; PHT = prehypertensive; SBP = systolic blood pressure; SHT = severely hypertensive; UC = urinary creatinine; UP = urinary proteins; UPC = urinary protein-to-creatinine ratio. * SBP was not available in 6 cats at risk, 1 cat at IRIS stage 1, 10 cats at IRIS stage 2, 1 cat at IRIS stage 3 and 1 cat at IRIS stage 4.

**Table 4:** Urinary big endothelin-1 concentrations (bigET-1) and urinary big endothelin-1 to urinary creatinine ratio (bigET-1:UC) in samples of cats classified in the different stages and substages of the International Renal Interest Society (IRIS) classification. Samples collected at the time of inclusion and at the different checkpoints were included. Number and percentage (in brackets) of cases included in each group are also reported.

		bigET-1 (pg/mL)	bigET-1:UC
	*n* (%)	Median (Range)	Median (Range)
At risk	21 (35%)	1.8 (0.0–34.3)	0.9 (0.0–13.2)
CKD (IRIS 1–4)	39 (65%)	3.1 (0.0–50.7)	1.9 (0.0–55.6)
IRIS 1	9 (15%)	0.0 (0.0–38.9)	0.0 (0.0–26.4)
IRIS 2	21 (35%)	1.1 (0.0–50.7)	0.4 (0.0–29.4)
IRIS 3–4	9 (15%)	10.0 (4.0–34.6)	21.5 (2.3–55.6) *
NP	38 (63.3%)	1.0 (0.0–50.7)	0.3 (0.0–55.6)
BP	14 (23.3%)	3.4 (0.0–38.9)	1.7 (0.0–26.4)
P	8 (13.3)	14.1 (2.5–15.3)	11.0 (3.0–46.4) ^†^
SBP < 160 mmHg	33 (75%)	4.9 (0.0–50.7)	1.6 (0.0–55.6)
SBP ≥ 160 mmHg	11 (25%)	3.1 (0.0–16.5)	2.5 (0.0–30.2)

BP = borderline proteinuric; CKD = chronic kidney disease; IQR = interquartile range; NP = non proteinuric; P = proteinuric; SBP = systolic blood pressure. * *p* = 0.001 vs. “At risk”, IRIS 1 and IRIS 2 ^†^ *p* = 0.029 vs. NP.
